# The Stimulating Effect of Bright Light on Physical Performance Depends on Internal Time

**DOI:** 10.1371/journal.pone.0040655

**Published:** 2012-07-11

**Authors:** Thomas Kantermann, Sebastian Forstner, Martin Halle, Luc Schlangen, Till Roenneberg, Arno Schmidt-Trucksäss

**Affiliations:** 1 Centre for Behaviour and Neurosciences, University of Groningen, Groningen, The Netherlands; 2 County Hospital Schrobenhausen, Schrobenhausen, Germany; 3 Department for Prevention and Sports Medicine, Technical University Munich, Munich, Germany; 4 Philips Lighting, Eindhoven, The Netherlands; 5 Institute for Medical Psychology, Ludwig-Maximilians-University Munich, Munich, Germany; 6 Institute of Exercise and Health Sciences, University of Basel, Basel, Switzerland; Universidad Europea de Madrid, Spain

## Abstract

The human circadian clock regulates the daily timing of sleep, alertness and performance and is synchronized to the 24-h day by the environmental light-dark cycle. Bright light exposure has been shown to positively affect sleepiness and alertness, yet little is known about its effects on physical performance, especially in relation to chronotype. We, therefore, exposed 43 male participants (mean age 24.5 yrs ± SD 2.3 yrs) in a randomized crossover study to 160 minutes of bright (BL: ≈ 4.420 lx) and dim light (DL: ≈ 230 lx). During the last 40 minutes of these exposures, participants performed a bicycle ergometer test. Time-of-day of the exercise sessions did not differ between the BL and DL condition. Chronotype (MSF_sc_, mid-sleep time on free days corrected for oversleep due to sleep debt on workdays) was assessed by the Munich ChronoType Questionnaire (MCTQ). Total work was significantly higher in BL (median 548.4 kJ, min 411.82 kJ, max 875.20 kJ) than in DL (median 521.5 kJ, min 384.33 kJ, max 861.23 kJ) (p = 0.004) going along with increased exhaustion levels in BL (blood lactate (+12.7%, p = 0.009), heart rate (+1.8%, p = 0.031), and Borg scale ratings (+2.6%, p = 0.005)) in all participants. The differences between total work levels in BL and DL were significantly higher (p = 0.004) if participants were tested at a respectively later time point after their individual mid-sleep (chronotype). These novel results demonstrate, that timed BL exposure enhances physical performance with concomitant increase in individual strain, and is related not only to local (external) time, but also to an individual’s internal time.

## Introduction

Performance and alertness vary over the course of the day and depend on both time awake (homeostatic sleep pressure) and phase of the internal circadian clock [1], [2]. The circadian clock is synchronized (entrained) by zeitgebers to the 24-hour day. The light-dark cycle is by far the most potent zeitgeber [3], [4], [5], with light at around dawn shortening the internal cycle length and light at around dusk lengthening the internal cycle length [6], [7], [8], [9]. Individuals can differ substantially in the phase relationship between their internal clock time and external local time, a phenomenon called chronotypes [10].

In addition to light being a zeitgeber for the circadian clock [8], [11], [12], it can also directly affect levels of alertness and sleepiness. This acute effect of light depends on time-of-day of light exposure, on light intensity, duration, spectral composition, and on an individual’s light history [1], [13]. Yet, only little is known about light effects on physical performance [2]. Several studies tested physical performance under different light exposures (ranging from 50 lx to more than 6.000 lx) and at different times of day and found no significant differences [14], [15], [16].

Both the effects of light and physical performance depend on when individuals are tested in reference to their internal time (chronotype). Thus, if chronotype is neither considered in the study design nor in the analysis, interpretations of the results are questionable (notably, none of the prior studies considered chronotype). We, therefore, applied a crossover protocol to evaluate the effect of bright light (BL ≈ 4.420 lx) compared to dim light (DL ≈ 230 lx) on physical performance, in relation to the participant’s chronotype. Besides measuring total work (kJ), we recorded levels of blood lactate, heart rate (HR), body temperature, oxygen uptake, carbon dioxide expiration, perceived exertion (Borg scale), and subjective motivation and light acceptance. In our study, we tested the following two hypotheses: (i) performance as well as the outcome of the concomitant measures are influenced by light levels (BL versus DL); (ii) the strength of these effect depends on chronotype.

## Materials and Methods

The study took place between April 2007 and August 2007 at the Department of Prevention and Sports Medicine of the Technical University of Munich (ethical approval was granted from the Technical University of Munich Ethics Committee). Trial has not been registered in Clinical Trials because intervention was not used to modify a health outcome. For recruitment of male participants, flyers and letters were distributed at the Technical University of Munich and its student dorms. Inclusion criteria were: age 20–30 years; good general health; no diagnosis of skin-, eye- or psychiatric diseases; no medication interfering with photosensitivity; no current shift-work; no use of tanning lights; no time-zone travels for four weeks prior and during the study. Written informed consent was collected from all participants at the beginning of the study. At baseline, the following measures have been collected from all participants: height (cm), weight (kg), body mass index (kg/m^2^), body fat content (%), and waist-to-hip ratio. All participants completed a daily sleep diary to assess their sleep duration seven days before each time trial with either BL or DL exposure. Participants have not been compensated for study participation. Initially, a total of 44 participants were recruited. One participant had to be excluded because of upper respiratory airway infection after the initial ergometer test (see below), leaving 43 participants for the final study.

Bicycle ergometer test. Initial workload for the ergometer sessions was determined by a step test on a bicycle ergometer (Sport Excalibur, Lode Medical technology, Groningen, The Netherlands) one week before the study, starting at 50 W with 25 W increments every three minutes until exhaustion (defined as respiratory exchange ratio, RER >1.0, Borg ≥18, unable to keep pedaling frequency >60 rpm). Gas analysis was made with ZAN metabolic cart (ZAN 600 USB CPX, nSpire Health GmbH, Germany). VO_2_ and VCO_2_ were measured every 10 seconds and peak oxygen uptake (VO_2peak_) was defined as the highest oxygen uptake reached during the testing. Heart rate was measured with a 12-lead ECG, and lactate was determined in capillary blood samples (20 µl) with Biosen 5040 or Biosen C-line (EKF Diagnostic, Germany) taken in the third minute of every step. Individual anaerobic threshold (IAT) was determined by standard software (Ergonizer Software, Version 2.5.9, Freiburg, Germany). Before the test session on the same cycle ergometer study participants had a warming up phase of 10 minutes at 40% IAT. After that participants made a 40 minutes test ride. Intensity was set at the level of the IAT of the step test, providing the same pedaling frequency of the step test. If the cadence increased, the workload increased and vice versa. The workload (P) changed quadratically (factor α) with pedaling cadence (C) according to the formula:
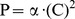



During test sessions, participants could choose any pedaling frequency above 70 rpm. Gases and heart rate were continuously recorded, and RPE and lactate were assessed every four minutes. Workload was continuously monitored and stored on a PC for analysis. Participants were advised to achieve as much work as possible over the 40-minute session. Time between the two sessions was one week. Between sessions, participants were advised to follow their normal daily routine and to abstain from non-habitual physical exercising. Even habitual intensive training was prohibited two days prior to each session, to control for confounding effects of prior exercise on the performance measurements during the test sessions. Participants completed a daily training diary to control for study compliance.

### Bright Light and Dim Light Exposure

**Figure 1:**
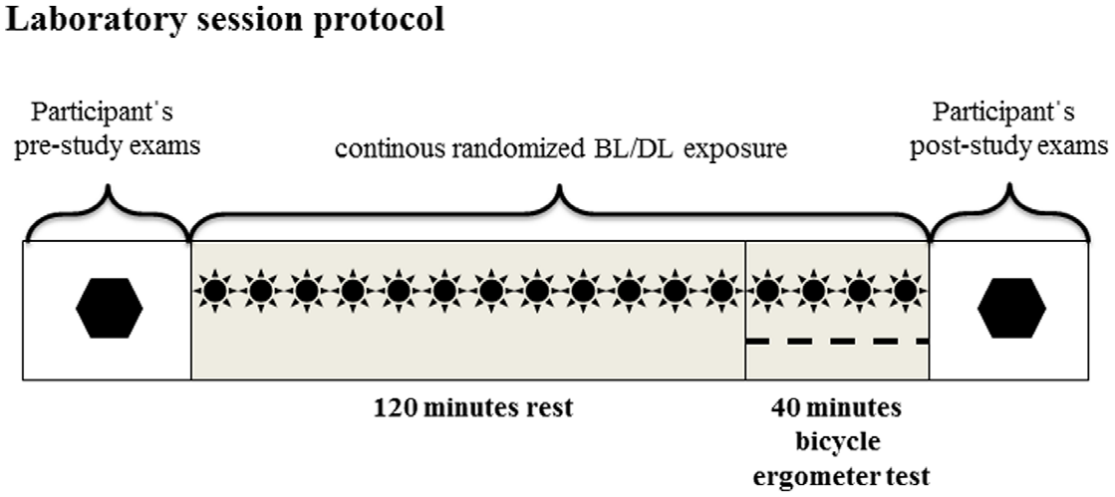
Laboratory session protocol: sun  =  continuous randomized bright/dim light exposure; dashed line  =  measurement of workload, heart rate, oxygen uptake, blood lactate, Borg scale ratings, body temperature; hexagon  =  Participant’s pre-study and post-study assessment of subjective light acceptance and Situational Motivation Scale (SIMS) ratings each prior and after the overall 160 minutes light exposure and 40 minutes bicycle ergometer test session.

For seven days prior and on the days between the two light exposure sessions, participants were asked to wear dark sunglasses (without further specification) when outdoors to shield the eyes from sunlight, and to avoid interference with the artificial light exposure (BL and DL) on the study days. To control for study compliance, participants completed daily diaries on their time-spent-outdoors (as an approximation for outdoor light exposure). It was not feasible to collect valid information about the times and frequency participants have worn their sunglasses. No further control of outdoor light exposure has been performed. The study room was air-conditioned (air temperature 20 C°, air humidity 50%). The two experimental lights (HF3309 PL-L 36 W Philips EnergyLights, with a correlated color temperature of 5000 K) – placed at 60 cm distance in the participant’s direction of gaze – were the only light sources in the study room. In a randomized crossover study, 43 male participants were exposed to 160 minutes of bright light (BL: ≈4.420 lx, with 3.91^19^ photons/m^2^ and 1.42^01^ W/m^2^ in 380–740 nm light spectrum range) and dim light (DL: ≈230 lx, with 2.03^18^ photons/m^2^ and 7.38^01 ^W/m^2^ in 380–740 nm light spectrum range). Illuminance levels were measured at eye level vertically in the direction of gaze (e.g., facing the walls) using a Lux meter (product number 025495, Lichtmesstechnik GmbH Berlin). Control conditions are always difficult in experiments testing light effects. Participants were therefore informed that “special light” (with four red LEDs visible as low intensity red dots in the light emitting area of the device) was used in the DL condition. During the last 40 minutes of each light exposure session (BL or DL), participants were subjected to a bicycle ergometer test. The total achieved work (kJ), blood lactate, heart rate (HR), body temperature by infrared ear thermometry, oxygen uptake, and carbon dioxide expiration were measured, in addition to subjective ratings of perceived exertion (using the 6 to 20 point Borg scale [17]), subjective light acceptance (using 10 cm visual analogue scales asking, for example, to rate the lighting from “normal to bright”, from “drowsy to activating”, or from “interferes with reading to supports reading”), and completion of the Situational Motivation Scale (SIMS [18]). Bicycle ergometer test starting times for all 43 participants were between 4∶30 p.m. and 10∶26 p.m. with a median 5∶30 p.m. for BL and 5∶31 p.m. for DL. There was no significant difference in the distribution of the starting times between the BL and DL sessions. Figure 1 illustrates the laboratory session protocol.

### Grouping by chronotype

Chronotype (MSF_sc_, mid-sleep time on free days corrected for oversleep due to sleep debt on workdays) was estimated using the Munich Chronotype Questionnaire (MCTQ), and used as a reference for internal time [10] (the local times of each session were converted to hours since MSF_sc_ on an individual basis). For 36 of the 43 participants, the internal onset time of BL and DL light exposure was identical. Final analysis was performed only on these 36 participants. Based on their internal time at the onset of the light exposure, participants were separated into two equally sized groups – ‘earlier’ and ‘later’. The ‘earlier‘ group was on average tested 11.78 h after their MSF_sc_ (BL: mean 11.81 h; DL: mean 11.75 h), while the ‘later’ group was on average tested three hours later in reference to their MSF_sc_ (mean 14.79 h, with BL: mean 14.83 h; DL: mean 14.75 h). Since both sessions (BL and DL) were scheduled at the same local time point for each participant, the two groups (‘early’ and ‘late’) also reflect the participants’ chronotype: earlier chronotypes (‘larks’) show a larger time difference between their MSFsc and their actual local time point of the test sessions and hence they fall into the ‘later’ group, and later chronotypes (‘owls’) show a shorter time difference between their MSFsc and time point of the test sessions and therefore they fall into the ‘earlier’ group.

### Statistics

Data analysis was performed using SPSS versions 16.0 and 17.0 for Windows (SPSS Inc., Chicago, USA) and PASW Statistics 18.0 for Macintosh (IBM, Somers NY, USA). Shapiro-Wilk tests have been applied to test for normal distribution of the differences in the measurements performed in bright light (BL) and dim light (DL) condition. Normality of differences between these measurements in BL and DL was assumed since p-values of Shapiro-Wilk-tests were equal to 0.5 or above 0.05. Therefore, paired t-tests have been applied. Wilcoxon signed rank test were applied if differences were not normally but at least symmetrically distributed. The sign test, as a universally applicable test, has been applied to test whether median (not mean) of differences were non-zero at population level. In addition, mixed linear model adjusting for potential sinusoidal diurnal pattern has been applied. To detect an increase in total work (kJ) of 3% at a two-sided significance level of 5% and with statistical power of 80%, a total sample size of 34 participants was calculated.

## Results

The mean age (± SD) and the means (± SD) of the additional anthropometric and ergometric data for all 43 participants are shown in Table 1. The mean age of the final 36 male participants was 24.5 yrs ± SD 2.4 yrs (Table 2). There was no significant difference between the “earlier” group and the “later” group in age, height, weight, body mass index (BMI), body fat, waist-hip-ration (WHR), IAT (in both total watts and watts/kg body weight), heart rate at IAT, and VO_2_ peak (Table 2). There was neither a significant difference in sleep duration (assessed by daily sleep diaries) among the participants during the study period (data not shown) nor in self-reported time-spent-outdoors between the session days (as an approximation for outdoor light exposure; median time-spent-outdoors: 30 min; 0–270 min prior to BL sessions; 0–330 min prior to DL sessions). The number of participants that (based on self-reports) did wear sunglasses on the days of BL and DL exposure was not significantly different with N = 11 and N = 10, respectively. The participants’ SIMS-scores neither showed a significant difference for the comparison between ‘prior and after light exposure’ nor for the comparison between ‘dim and bright light exposure’. There were no reports by the participants of any adverse effects due to the exposure to the experimental lights.

**Table 1:** Baseline anthropometric and ergometric data of all study participants (N = 43).

Parameters	Mean (± SD)	Minimum	Maximum
**Age (years)**	24.5 (2.3)	20	29
**Height (cm)**	180.6 (7.3)	163	196
**Weight (kg)**	77.1 (8.9)	61	94.5
**Body mass index (kg/m^2^)**	23.6 (1.8)	19.4	27.9
**Body fat (%)**	12.4 (3.1)	7.6	20
**Waist-to-hip ratio**	0.97 (0.03)	0.89	1.01
**IAT (Watts)**	218 (46)	146	335
**IAT (Watts/kg)**	2.9 (0.5)	1.9	4.3
**Heart rate at IAT (1/min)**	159.6 (12.5)	130	184
**VO_2_ peak (ml/kg/min)**	57.9 (6.2)	41.3	71

SD  =  standard deviation.

**Table 2:** Baseline anthropometric and ergometric data of the “earlier group” (N = 18), “later group” (N = 18) and of both groups combined (N = 36).

	“Earlier group”	“Later group”	Both groups combined
Parameters	Mean (± SD)	Mean (± SD)	Mean (± SD)
**Age (years)**	24.3 (2.4)	24.8 (2.5)	24.5 (2.4
**Height (cm)**	182 (7.8)	178.1 (6.4)	180 (7.3)
**Weight (kg)**	78.5 (8.3)	75.3 (8.4)	76.9 (8.4)
**Body mass index (kg/m^2^)**	23.6 (1.3)	23.7 (1.7)	23.7 (1.5)
**Body fat (%)**	12.7 (3.4)	12.2 (2.4)	12.4 (2.9)
**Waist-to-hip ratio**	0.97 (0.03)	0.97 (0.03)	0.97 (0.03)
**IAT (Watts)**	220.4 (46.4)	212.4 (52.6)	216.4 (49)
**IAT (Watts/kg)**	2.8 (0.5)	2.8 (0.6)	2.8 (0.5)
**Heart rate at IAT (1/min)**	156 (8.9)	161 (15.2)	158.4 (12.5)
**VO_2_ peak (ml/kg/min)**	57.6 (6.3)	57.9 (5.5)	57.7 (5.9)

No significant differences between “earlier group” and “later group” in any of these parameters (one-way ANOVA). SD  =  standard deviation.

Total work was significantly higher in bright light (BL; median 548.4 kJ, min. 411.82 kJ, 25% quartile 494.52 kJ, 75% quartile 610.90 kJ, max. 875.20 kJ) compared to dim light (DL; median 521.5 kJ, min. 384.33 kJ, 25% quartile 470.49 kJ, 75% quartile 617.01 kJ, max. 861.23 kJ) at the end (p = 0.004). This was even true for every single time point in each session (p≤0.004; all paired t-tests).

**Figure 2:**
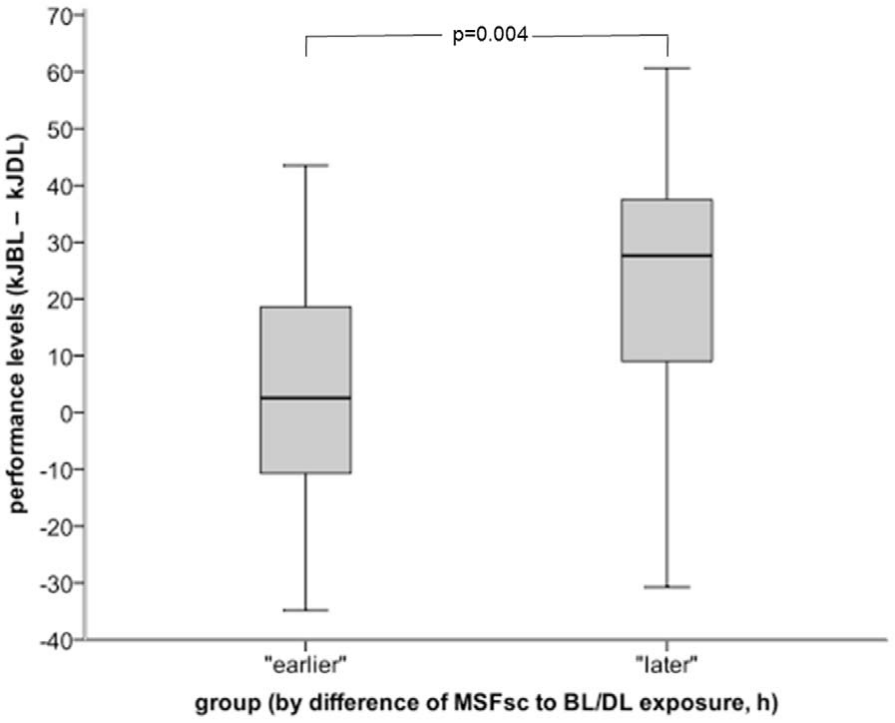
Difference in cumulative total work expressed in kilo Joule (kJ) during the bicycle ergometer tests for the “later group” (N = 18) and the “earlier group” (N = 18). See Methods for grouping details. Total work was significantly higher in the “later group” (p = 0.004, paired t-test).

Total work during both BL and DL sessions (kJ_BL_ – kJ_DL_) was significantly higher in the ‘later’ group compared to the ‘earlier’ group (p = 0.004, paired t-test; Figure 2). Compared to DL, BL lead to an additional work of 27.6 kJ (min. −30.7 kJ, 25% quartile 8.7 kJ, 75% quartile 37.7 kJ, max. 60.7 kJ) in the ‘later’ group, while BL increased the work by only 2.6 kJ (min. −34.8 kJ, 25% quartile −13.2 kJ, 75% quartile 19.1 kJ, max. 43.5 kJ) in the ‘earlier’ group (not significant). To test for possible confounding that later chronotypes (‘owls’) might have been tested at a later local time point and that earlier chronotypes (‘larks’) might in turn have been tested at an earlier time point, we applied parametric correlation analysis. There was no significant correlation between the 36 participants’ chronotype (MSFsc) and time point of test in either BL (bright light) or DL (dim light) condition (data not shown).

To assess the effects of BL irrespective of internal time, we analyzed all 43 participants for clinical parameters. BL significantly increased levels of blood lactate (mean lactate level in BL: 5.47 mmol/l ± SD 1.49; mean increase above DL: 0.62 mmol/l ± SD 1.46; p = 0.009, paired t-test), HR (mean HR in BL: 168 bpm ± SD 12; mean increase above DL: 3 bpm ± SD 8; p = 0.031, paired t-test), and Borg scale ratings (perceived exertion) (mean ratings in BL: 15.7± SD 1.1; mean increase above DL: 0.4± SD 0.9; p = 0.005, with both paired t-test and sign test). Applying a mixed linear model to adjust for potential sinusoidal diurnal pattern (time-of-day effect) in the measurements did not significantly change these results (data not shown).

## Discussion

Non-invasive measures to increase physical performance at an individual’s optimal time-of-day is of major interest for a wide range of applications, from athletic competitions [15] to shift-work settings [19]. Controlled (bright) light exposure has been clearly shown to affect circadian entrainment [6] and to significantly increase performance [20]. Here, we show for the first time that bright light also increases physical performance (using a bicycle ergometer). This increase is accompanied by elevations of HR, blood lactate and rate of subjectively perceived exertion on the Borg scale indicating a higher level of individual strain. Notably, these results did not change after applying a mixed linear model to adjust for potential sinusoidal diurnal pattern ( = time-of-day effect) in our measurements.

In contrast to our results, O’Brien and O’Conner [15] found no effects of light (250–6434 lx) on bicycle ergometer performance. Our study differs, however, in several aspects. While in our design light exposure started 2 h prior to and continued during the 40-min cycling session, O’Brien and O’Conner exposed their subjects only during their 20 min cycling sessions to light. In addition, they performed their tests at very different local times (8∶00–18∶00) and did not consider internal time of their subjects. As physical performance has been reported to peak in the late afternoon/early evening [2], we studied the differential effects of bright light, depending on individual internal time.

Our results not only show that bright light increases physical performance levels (kJ) but also that this effect depends on individual internal time. BL-induced increments were significantly higher in sessions scheduled approximately 14.5 h after individual mid-sleep (MSF_sc_; chronotype) than three hours before. The internal-time-specific BL-effect may have reasons beyond the times of exercising, such as, for example, a stress-induced activation of the sympathetic nervous system. However, Monteleone and colleagues [21] did not find that exercising on an ergometer bicycle in the evenings and early part of the night leads to stress-induced sympathetic activation, whereas they did observe such activation for the same test performed in the second half of the night [22]. We advised participants to avoid any intensive training two days prior each session, to control for confounding effects on the actual performance measurements during the study.

One could argue that the time of year this study was performed (April to August) includes significant changes in day length (photoperiod), possibly changing circadian phase of entrainment or that the cycling sessions and the 160-min bright light exposures affected internal time. Although we did not measure circadian phase objectively (e.g. by melatonin samples), we exclude these possibilities for several reasons: first, the random crossover design controls for season effects (in addition, our participants spent only little time outdoors); second, while phase of entrainment changes in spring until approximately end of March, it remains remarkably stable during the summer months [23] (sleep duration and sleep timing of our participants remained stable throughout the study period); finally, controlled studies have shown that physical performance has little effect on circadian phase [24], [25].

Several studies have shown positive effects of BL on alertness [for review, see 1]. We also found that BL was perceived subjectively as ‘activating’ and ‘aggressive’, which might partially increase total work in all subjects regardless of the internal zeitgeber (data not shown). Indeed, all parameters reflecting height of physical strain (i.e. heart rate, lactate) were higher in BL. Interestingly, later chronotypes (‘earlier’ group) indicated to be ‘more motivated’ in DL than in BL, whereas it was the later chronotypes that rated the BL to be more ‘supportive for reading’ (Participants were allowed to bring something to read during the 120 minutes of light exposure prior the 40-minute ergometer test. The reading material did not obstruct the view towards the experimental lights and hence did not influence illuminance at eye level of the participants. This is in agreement with the result that participants in the “later group” (hence rather earlier chronotypes, therefore having a longer temporal difference between their MSF_sc_ and respective light exposure) had the highest benefit from BL, and reported being more ‘motivated’ in BL (data not shown). In addition, participants in the “later group” (earlier chronotypes) probably had a higher homeostatic sleep pressure as they have been awake for a longer duration at the time point of their test sessions [3], [26], and therefore may be affected by the BL more strongly. However, there was no change in sleep duration (assessed by using daily sleep diaries) across the study period, and one can only speculate on any such effect on the homeostatic sleep pressure here.

Although more studies are needed for a more detailed cost/benefit estimation of different BL interventions on physical performance, our results indicate that we will in future be able to recommend tailored and optimally timed training sessions for individuals with different chronotypes.
